# The Half Year

**Published:** 1884-07

**Authors:** 


### THE HALF YEAR.

In this number the series of articles by Dr. Miller upon "Fermentation in the Human Mouth" is brought to a close. They have attracted wide-spread attention, and form a valuable contribution to the permanent literature of the profession. Every dentist who pretends to keep pace with the advance of thought in his chosen field should become familiar with them. That this opportunity may be afforded all, we have had them reprinted in a handsome pamphlet form, and the complete series may be procured at the office of the INDEPENDENT PRACTITIONER for fifty cents per copy.

There is another way in which the work may be obtained. We will send a copy to every new subscriber to this journal, and we could not offer a better premium. Dr. Miller will continue his contributions, and all his original work will be given to the profession through the INDEPENDENT PRACTITIONER, for he is now one of its proprietors and publishers. The record the journal has made during the year in which it has been under its present management is a guarantee of what the coming volume will be, although we think that added experience and increased facilities will enable us to do better in the future than we have in the past. Every dollar that is sent us for subscriptions will be expended in making the journal better. May we not, then, ask its friends to do what they can to obtain for it new subscribers? All subscriptions should commence with the January or July number.

